# Offensive Feet

**Published:** 1887-11

**Authors:** Edwin DeBaun

**Affiliations:** Passaic, N. J.


### OFFENSIVE FEET.

Edwin deBaun, M. D., Passaic, N. J.

On January 2d, 1886, there came to me a young woman, twenty-two years of age, blonde and hysterical, complaining that her feet perspired profusely, and that the odor was so penetrating and offensive that she could not sit in the room with any one more than a few minutes at a time, when the odor would permeate the whole apartment. The odor was almost indescribable, resembling, if anything, carrion.

The foot-sweat was confined only to the heels, in such a manner that should one arm of a compass be placed on the middle posterior part of the heel and a circle of one inch in diameter be described it would exactly outline the diseased part.

The line of demarkation was so plain as to be easily perceptible, both by sight and touch.

The diseased part had a whitish appearance, changing at times to a bluish and sometimes a reddish hue, and was shriveled as if the heels had been held in water for a long time, the remainder of each foot being perfectly healthy. The sensation experienced was simply that of numbness.

Her occupation compelled her to stand upon her feet, handling freshly manufactured rubber. She had been to several physicians and had tried all manner of washes and ointments for two years, but to no avail, her condition growing steadily worse. Together with the foot symptoms, her face would break out with little pustules regularly each month at the menstrual period, and her general health was poor. I gave her Baryta carbonica$^{3x}$ morning and evening, and instructed her to bathe her feet twice daily with warm water and Castile soap, to wear fresh, clean stockings each day, and to have two pair of shoes, wearing them upon alternate days. I saw her again in a week, when she reported marked improvement, and at the expiration of five weeks discharged her cured. Six months later she called at my office and said she had not had a return of the foot trouble, and that the pustular condition of the face had entirely disappeared and her health was improved in every respect.

